# Nutrition Counseling Frequency and Telehealth Integration on Cardiometabolic Outcomes in Metabolic Syndrome: A Randomized Controlled Trial in Turkey

**DOI:** 10.3390/medicina61122124

**Published:** 2025-11-28

**Authors:** Kübra Yıldız-Güler, Miraç Vural-Keskinler, Mehmet Fisunoğlu

**Affiliations:** 1 Department of Nutrition and Dietetics, Faculty of Health Sciences, Istanbul Medeniyet University, 34862 Istanbul, Turkey; 2 Department of Internal Medicine, School of Medicine, Istanbul Medeniyet University, 34700 Istanbul, Turkey; miracvural@hotmail.com; 3 Department of Nutrition and Dietetics, Faculty of Health Sciences, Hacettepe University, 06100 Ankara, Turkey; fisunogl@hacettepe.edu.tr

**Keywords:** metabolic syndrome, abdominal obesity, diet therapy, nutrition counseling, telehealth, randomized controlled trial, Turkey

## Abstract

*Background and Objectives:* As the demand for dietitian services continues to grow in high-volume healthcare settings, optimizing both the frequency and modality of follow-up has become crucial for sustaining and enhancing the effectiveness of medical nutrition therapy (MNT). This study evaluated a stepwise intensification model ranging from quarterly in-person visits to monthly in-person visits and weekly telehealth follow-ups in metabolic syndrome (MetS). *Materials and Methods:* Ninety-three patients with MetS were randomized into three groups based on the intensity of dietary consultations: the intervention group (IG, *n* = 31), the intensive intervention group (IIG, *n* = 30), and the control group (CG, *n* = 32). IG received four in-person visits (baseline and weeks 4, 8, and 12); IIG received the same four visits plus weekly telehealth calls; and CG received two at baseline and week 12. Nutrient intake, anthropometric and biochemical measurements were compared across groups. A regression model was applied to examine associations between the frequency of nutritional counseling and MetS components. *Results:* Higher counseling frequency was associated with greater improvements in dietary intake, anthropometric, biochemical and blood pressure parameters. Both intervention groups achieved greater reductions in waist circumference (*p* < 0.001), BMI (*p* < 0.001), and body weight (*p* = 0.002) than CG, with the IIG improvements in FPG (*p* < 0.001, η^2^ = 0.215) and blood pressure benefits (systolic: *p* < 0.001, η^2^ = 0.276; diastolic: *p* < 0.001, η^2^ = 0.148). In multivariable regression, counseling frequency independently predicted improvement in MetS diagnostic criteria (F = 9.395; *p* < 0.001, adjusted R^2^ = 0.313). *Conclusions:* These findings highlight that integrating telehealth into MNT for MetS may enhance its effectiveness while minimizing the burden on healthcare systems.

## 1. Introduction

Abdominal obesity, insulin resistance, diabetes, elevated blood pressure, and dyslipidemia collectively constitute metabolic syndrome (MetS), a cluster of cardiometabolic risk factors [1,2]. The prevalence of MetS is estimated at 24.3% in Europe, whereas it is higher in Türkiye at 32.9% [3,4]. The substantial global variation in prevalence is largely attributed to differences in diagnostic criteria used across studies. To standardize the definition, a joint consensus was established by the International Diabetes Federation Task Force on Epidemiology and Prevention, the National Heart, Lung, and Blood Institute, the American Heart Association, the World Heart Federation, the International Atherosclerosis Society, and the International Association for the Study of Obesity [2].

The increase in MetS prevalence from 37.6% in the 2011–2012 US National Health and Nutrition Examination Survey to 41.8% in recent estimates underscores a rising trend [5]. The individual components of MetS are highly responsive to lifestyle modifications, with medical nutrition therapy (MNT) widely recognized as one of the most effective and cost-efficient interventions [6,7]. Nutritional strategies are widely implemented as first-line approaches in the prevention and management of abdominal obesity [8,9], hyperglycemia [6,10], dyslipidemia [7], and hypertension [11]. As a structured dietary intervention, MNT is most effectively delivered by registered dietitian nutritionists, who are specifically trained in chronic disease management. Evidence consistently demonstrates that registered dietitian nutritionist-led care improves cardiometabolic outcomes, and recent perspectives highlight the importance of incorporating dietitians into primary care teams to ensure sustainable lifestyle change and optimize obesity management [12,13,14]. While its clinical benefits are well documented, the effectiveness of MNT depends heavily on the quality and continuity of dietitian–patient interactions. The American Diabetes Association recommends sixteen nutrition counseling sessions involving MNT (45–90 min each) within six months, and similarly, the Academy of Nutrition and Dietetics advises weekly or biweekly visits during early treatment [15].

In real-world settings, however, particularly in public hospitals, consultations are often restricted to 10–15 min and scheduled at three-month intervals due to limited resources. This discrepancy between recommended and actual follow-up frequencies may hinder adherence and reduce the long-term effectiveness of nutritional therapy. To address these practical limitations—including limited consultation time, high patient volume, and insufficient healthcare staff—alternative strategies have been explored to increase the frequency and continuity of nutritional care. Telehealth has emerged as a promising solution in this context. The American Diabetes Association and AHA/NHLBI recommends integrating telehealth capabilities into diabetes and cardiovascular care to improve access and support patient management [16,17].

Among nutritional interventions for weight management, hybrid models combining in-person visits with telehealth modalities such as phone or video calls, text messages, and emails are increasingly used in clinical practice. Telehealth became an integral part of clinical practice during the COVID-19 pandemic and has continued to expand its role beyond the pandemic [18]. A telehealth program providing educational content and follow-up both in-person and remotely was shown to improve adherence to a heart-healthy diet, increase physical activity, and promote weight loss in young adults [19]. These hybrid models have demonstrated potential to enhance outcomes by supporting behavioral change and improving adherence to lifestyle recommendations [20,21].

Despite growing evidence on both MNT and telehealth, limited research has investigated how varying follow-up intensities, particularly in hybrid models, influence the comprehensive cardiometabolic profile of individuals with MetS. This gap highlights the need for innovative approaches. Therefore, this study aimed to examine the effects of progressively intensified nutrition counseling frequency, incorporating telehealth into MNT, on cardiometabolic outcomes in individuals with MetS. To our knowledge, it represents the first randomized controlled trial to simultaneously evaluate both increased in-person consultation frequency and telehealth integration within the same intervention model.

## 2. Materials and Methods

### 2.1. Study Setting and Design

This randomized, controlled, three-arm parallel-group trial was conducted at the Diabetes, Hypertension, and Hyperlipidemia Outpatient Clinic of Göztepe Prof. Dr. Süleyman Yalçın City Hospital (Istanbul, Türkiye). Participants were randomly assigned to three groups differing in consultation intensity. CG received two in-person visits (baseline and week 12); IG received four (baseline and weeks 4, 8, and 12); and IIG received the same four in-person visits as IG, plus telehealth follow-ups between sessions, totaling 13 contacts.

Ethics approval was obtained from the Istanbul Medeniyet University Clinical Research Ethics Committee (approval no. 2022/0167; 30 March 2022). All participants provided written informed consent. The trial was retrospectively registered with ClinicalTrials.gov [NCT: 06813131].

### 2.2. Participants

All participants had a physician-confirmed diagnosis of MetS according to the joint interim consensus criteria. According to these criteria, although the cut-off values may vary depending on the population, the presence of at least three of the following conditions establishes the diagnosis of MetS increased waist circumference, fasting plasma glucose (FPG) level > 100 mg/dL, triglyceride (TG) level > 150 mg/dL, blood pressure with systolic ≥ 130 mmHg and/or diastolic ≥85 mmHg, and HDL-cholesterol level < 40 mg/dL in men or <50 mg/dL in women. [2]. Inclusion criteria also required age 18–65 years and HbA1c < 8.5%. Individuals were excluded if they were using lipid-lowering or antihypertensive agents; glucose-lowering medications other than metformin; dietary supplements known to affect metabolic parameters; had renal, hepatic, or immune system disease, cancer, type 1 diabetes; or were pregnant or lactating.

The sample size was calculated for the group-by-time interaction on waist circumference (WC) (3 groups, 4 time points; RM-ANOVA/LMM) using G*Power v3.1 (Heinrich-Heine-Universität Düsseldorf, Düsseldorf, Germany) [22]. A hierarchical gatekeeping approach was prespecified: the interaction was tested at α = 0.05, and if significant, the prespecified week-12 contrasts (IG vs. CG; IIG vs. IG) were evaluated. Based on variance observed in previous MetS interventions and a clinically meaningful 5.0 cm difference at week 12 with 90% power, 30 participants per group were required. Accounting for approximately 10% attrition, the target enrollment was set at 33 participants per group. Analyses of secondary outcomes (blood pressure, fasting glucose, HDL-C, and triglycerides) were exploratory and may not have been sufficiently powered to detect small-to-moderate effects.

**Figure 1:**
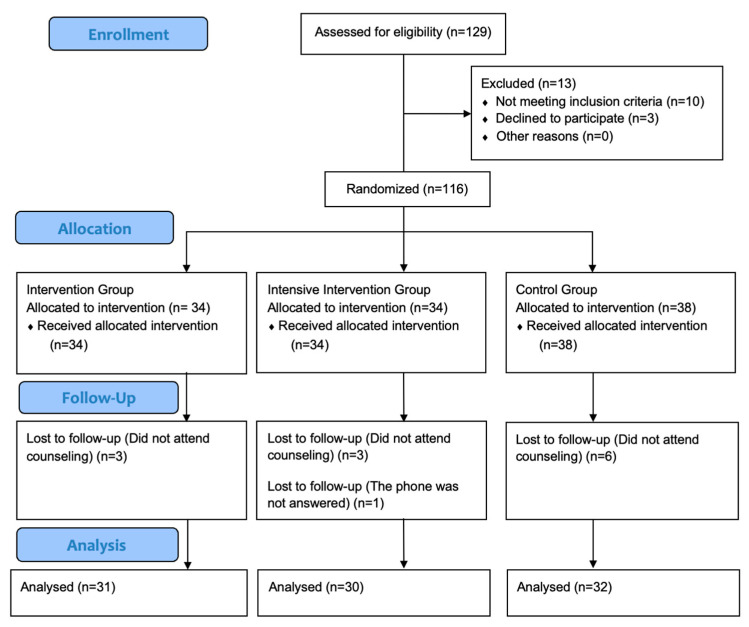
CONSORT flow diagram presents number of individuals screened; those excluded with reasons (not meeting inclusion criteria, declined to participate, other), randomized and allocated to intervention, intensive intervention or control groups; the number lost to follow-up with reasons, and the number included in the final analysis.

The study protocol was explained to all participants. The trial adhered to CONSORT 2010 guidelines [23]. Figure 1 presents the CONSORT flow diagram of screening, randomization, and analysis.

### 2.3. Intervention

The procedures conducted with the participants during the counseling are in the Table A1. As part of standard care, all individuals presenting to the outpatient clinic with a physician-confirmed diagnosis of MetS received the “Ministry of Health of the Republic of Türkiye Healthy Eating Brochure” along with brief physician-delivered counseling emphasizing the fundamental role of weight reduction and healthy eating in the management of MetS. Subsequently, patients were first referred by the physician to the clinical biochemistry laboratory where venous blood samples were collected and analyzed under standardized procedures for the measurement of fasting plasma glucose (mg/dL), insulin (mU/L), HOMA-IR, HbA1c (%), HDL-C (mg/dL), LDL-C (mg/dL), TG (mg/dL), hs-CRP (mg/L), AST (mg/dL), and ALT (mg/dL). Following biochemical testing, patients were directed to the clinic nurse for eligibility screening. Those confirmed as eligible were allocated to study groups through block randomization and subsequently referred to the study dietitian for MNT counseling. Participants were randomly assigned to one of three groups (CG, IG, IIG) using a centralized, web-based block randomization system (block size = 6) to ensure balanced allocation. The randomization sequence was computer-generated by an independent data coordinator who had no role in recruitment or assessment. After a physician confirmed the diagnosis of metabolic syndrome and participant eligibility, the clinic nurse enrolled participants and assigned them to groups according to the pre-generated sequence. The allocation sequence was concealed from both investigators and participants until assignment.

Although blinding of participants and investigators was not feasible due to the nature of the behavioral nutrition intervention, several measures were taken to minimize bias. Participants were only informed only about their own follow-up procedures, ensuring partial blinding. Anthropometric and blood pressure measurements were conducted by the study dietitian and clinic nurse trained in standardized protocols. Waist circumference was measured according to Word Health Organization (WHO) guidelines, and blood pressure was recorded with a calibrated automated device. Laboratory results were obtained from the hospital’s electronic records, and data were entered by an independent clerk not involved in the intervention or assessments.

### 2.4. Nutrition Counseling

Nutrition counseling sessions followed a standardized protocol and were conducted as follows: sociodemographic information was collected; physical activity was assessed; anthropometric and blood pressure measurements were obtained; dietary habits were assessed; dietary intake records were collected; and individualized MNT was planned.

Anthropometric measurements, including height, weight, and WC, were recorded using calibrated equipment. Body weight and body composition were measured by bioelectrical impedance analysis with an Omron Karada Scan BF511 device (Omron Healthcare Europe B.V., Hoofddorp, The Netherlands; resolution 0.1 kg). WC was measured according to WHO recommendations [24]. Body mass Index (BMI) was calculated (kg/m^2^) and classified per WHO criteria [25]. Blood pressure was measured on the right arm after 10 min of seated rest using a Beurer BM-26 (Beurer BM-26, Beurer GmbH, Ulm, Germany) device; participants with blood pressure ≥ 130/85 mmHg were classified as having elevated blood pressure [2].

Dietary intake was assessed using 24 h dietary recalls. Dietary data were entered into the Nutrition Information System (version 8.0) to derive macro- and micronutrient intakes.

Energy requirements were calculated using the Mifflin–St Jeor equation [26]. Total dietary energy intake was then prescribed at 500–700 kcal/day below estimated total energy expenditure [15,27]. Taking participants’ dietary habits and sociodemographic and sociocultural characteristics into account, individualized MNT targeted 50–60% carbohydrate, 10–20% protein, and 25–35% fat, with fatty acid composition goals of less than approximately 10–15% of energy from monounsaturated fatty acids (MUFA), less than 7–8% from polyunsaturated fatty acids (PUFA), less than 10% from saturated fatty acids (SFA), and less than 1% from trans fatty acids, along with 20–30 g/day dietary fiber and low sodium intake [28,29]. Participants were advised to avoid white bread, fried foods, processed meats, pickled/brined foods, and packaged snacks; to prefer whole-grain bread; to consume raw vegetables with meals; and to eat fish at least once weekly. Meal frequency was individualized to support appetite control, typically 4–6 meals/day [15,30].

At in-person follow-up visits (weeks 4 and 8), nutrition prescriptions were reviewed in light of updated anthropometry; if the change in estimated energy requirement exceeded ±100 kcal/day, the diet was recalibrated. Non-adherence—assessed by anthropometric trajectory or self-report—was not considered a withdrawal criterion; all participants were followed as planned, and the importance of dietary adherence for MetS management was emphasized at each visit. At the end of every visit, the next appointment was scheduled, and a telephone reminder was placed approximately one week before the planned date. In the IIG, weekly tele-nutrition calls were conducted on a fixed weekday throughout the 12-week study period. Each session lasted approximately 10 min and followed a structured protocol. The calls began with a brief greeting and assessment of the participant’s current status, followed by a 24 h dietary recall to monitor adherence to the prescribed nutrition plan. Participants received brief motivational counseling emphasizing the health benefits of dietary adherence, and any questions related to the nutrition prescription were addressed. These calls were designed to ensure consistent contact and support, and were conducted by the same study dietitian to maintain intervention fidelity. No validated dietary adherence scale was used; instead, adherence was monitored through self-reported dietary recalls obtained during the teleconsultations.

### 2.5. Statistical Analysis

Given its established association with cardiometabolic risk and other MetS components [31], WC was designated as the primary outcome. Secondary outcomes were FPG, HDL-C, TG and blood pressure measurements.

Depending on the distribution of the data, parametric or nonparametric methods were used. Group-by-time effects across baseline and weeks 4, 8, and 12 were evaluated using repeated-measures ANOVA with Bonferroni-adjusted post hoc tests. To control for Type I error, Bonferroni correction was applied for post hoc pairwise comparisons. No further correction across outcome domains was performed, as the analyses focused on a predefined set of primary (WC) and secondary (FPG, HDL-C, TG, and blood pressure) outcomes. Statistical analyses followed a per-protocol approach. Participants who discontinued the intervention had only baseline measurements and were therefore excluded from longitudinal analyses. This method was considered appropriate given the small number of dropouts and the absence of follow-up data for those who discontinued. Missing data were not imputed, as incomplete cases lacked repeated measures required for longitudinal modeling. Associations among dietary, anthropometric, and biochemical variables were examined using Pearson’s correlation, with r < 0.40 considered weak [32]. Linear regression analyses were conducted to assess the effect of consultation frequency on changes in MetS parameters. Analyses included only participants who completed follow-up in their assigned groups. A two-sided *p* < 0.05 was considered statistically significant. All statistical analyses were performed using IBM SPSS Statistics, version 25 (IBM Corp., Armonk, NY, USA).

## 3. Results

Baseline demographic and lifestyle characteristics by group are presented in Table 1; groups were comparable at baseline (all *p* > 0.05). A total of 116 participants were randomized into three groups: IG (*n* = 34), IIG (*n* = 34), CG (*n* = 38). All participants received the assigned intervention at baseline, but a total of 13 participants did not complete the 12-week follow up (IG: 3, IIG:4, CG: 6) due to missing counseling sessions (*n* = 12) or inability to be reach by phone (*n* = 1). Consequently, participants were included in the primary outcome analysis (IG: 31, IIG: 30, CG: 32). Recruitment and follow-up were conducted between October 2023 and July 2024. The study was completed as planned.

**Figure 2:**
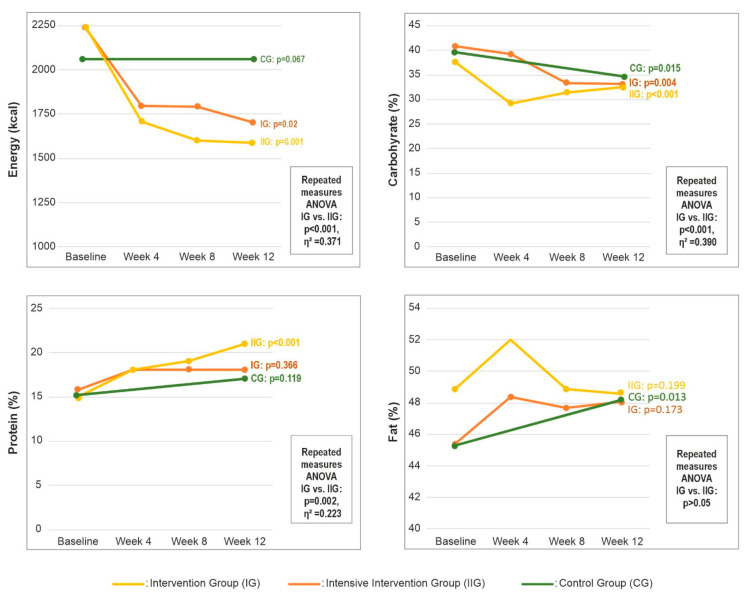
Changes in total energy intake and macronutrient distribution by group over 12 weeks. Abbreviations: CG—control group; IG—intervention group; IIG—intensive intervention group.

Figure 2 shows changes in total energy intake and the percentage of energy from carbohydrate, protein, and fat at baseline and weeks 4, 8, and 12. At baseline, energy and nutrient intakes did not differ between groups (all *p* > 0.05; Table A2). Within-group analyses showed that the percentage of energy from carbohydrate decreased from baseline to week 12 in all groups (IG, *p* = 0.004; IIG, *p* < 0.001; CG, *p* = 0.015) (Figure 2). In both intervention groups, total energy intake decreased (IG, *p* = 0.02; IIG, *p* = 0.001) (Figure 2), whereas fat as a percentage of energy increased in the CG (*p* = 0.013) (Figure 2). The IIG was the only group to show a significant increase in protein intake, together with reductions in SFA, MUFA, and PUFA intakes (all *p* < 0.05) (Table A2).

Repeated-measures ANOVA revealed statistically significant time effects for total energy intake and for carbohydrate, total fat, MUFA, PUFA, SFA and protein expressed as a percentage of energy (all *p* < 0.05). Effect size estimates indicated a large effect for changes in energy intake (η^2^ = 0.371) and carbohydrate intake (η^2^ = 0.390), a moderate effect for fat (η^2^ = 0.223), and a small effect for protein (η^2^ = 0.091) (Figure 2). Detailed statistics, including F values, degrees of freedom, and *p* values, are provided in Table A2.

Between-group comparisons of anthropometric outcomes showed that both intervention groups achieved significantly greater reductions in body weight (kg), BMI (kg/m^2^), with no significant differences between IG and IIG. Repeated-measures ANOVA demonstrated significant group-by-time interactions for all anthropometric outcomes (all *p* < 0.05). Effect-size estimates indicated large effects for changes in body weight (η^2^ = 0.541), a moderate effect for BMI (η^2^ = 0.291), and small effects for body-fat percentage (η^2^ = 0.120) and fat-free mass (η^2^ = 0.087). Full statistical results are provided in Table A3.

**Figure 3:**
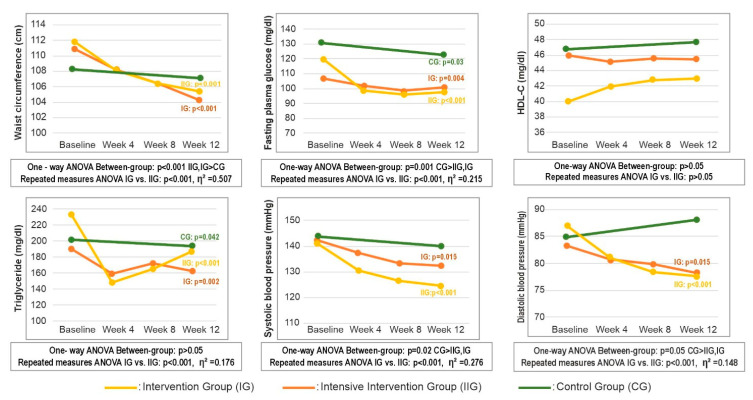
Change in metabolic syndrome parameters by group over 12 weeks. Abbreviations: CG—control group; IG—intervention group; IIG—intensive intervention group; HDL-C—high density lipoprotein cholesterol.

Figure 3 summarizes the 12-week changes in MetS parameters across the study groups. Both intervention groups achieved significantly greater reductions in WC from baseline to week 12 compared with the control group (all *p* < 0.001), with no significant difference between IG and IIG). Repeated-measures ANOVA confirmed significant group-by-time interaction for WC, with effect-size estimates indicating a large effect (*p* < 0.001, η^2^ = 0.507). Among biochemical parameters, FPG decreased significantly in all groups from baseline to week 12 (all *p* < 0.05), with the greatest reduction observed in the IIG (*p* = 0.009) (Table A3). TG also declined across all groups relative to baseline (Figure 3); group-by-time interactions between the two intervention arms indicated that consultation frequency exerted a large effect on TG (mg/dL) changes (*p* < 0.001, η^2^ = 0.176). For blood pressure, reductions in systolic (*p* < 0.001, η^2^ = 0.276) and diastolic (*p* < 0.001, η^2^ = 0.148) values were greater in the IIG compared with the IG (Figure 3).

Table A4 presents a more comprehensive panel of additional biochemical parameters measured throughout the 12-week study period. At baseline, no statistically significant between-group differences were observed for biochemical measures other than FPG (*p* = 0.021). Within-group analyses showed significant 12-week improvements in markers of glucose metabolism across all three groups: insulin, HOMA-IR, and HbA1c all decreased from baseline to week 12 (all *p* < 0.05; Table A4). For lipid metabolism, total cholesterol decreased in IG and CG, LDL-C decreased in IG (Table A4). Repeated-measures ANOVA revealed significant group-by-time interactions, with the largest effect observed for FPG (η^2^ = 0.215). Detailed statistics are provided in Table A4.

Table 2 presents correlations between consultation frequency and changes in MetS-related variables. Consultation frequency was weakly and negatively correlated with the percentage of energy from carbohydrate, and weakly and positively correlated with the percentages from protein and fat. For anthropometric outcomes, higher consultation frequency was associated with greater reductions in body weight, BMI, and WC. Similarly, weak positive correlations were observed between consultation frequency and improvements in FPG, insulin, systolic blood pressure and diastolic blood pressure. Change scores were calculated as baseline minus week 12; thus, positive values denote improvement over time. In multivariable linear regression with backward elimination, significant predictors of change in the number of MetS diagnostic criteria were identified; the final model explained 31.3% of the variance (adjusted R^2^ = 0.313) with no evidence of multicollinearity. Candidate variables were selected based on statistical significance in bivariate analyses (Table 3), clinical relevance to MetS, and responsiveness to dietary intervention, ensuring both statistical and practical interpretability.

## 4. Discussion

This parallel-group randomized trial compared three different frequencies of MNT over 12 weeks in adults with MetS. A clear gradient by consultation frequency emerged: higher consultation frequency was associated with clinically meaningful improvements in anthropometry (WC, the primary endpoint, body weight and BMI) as well as in glycemic and blood-pressure indices. Both intervention groups achieved larger reductions in WC, body weight and BMI than the CG. The IIG showed substantial decreases in FPG, systolic blood pressure and diastolic blood pressure than both the IG and CG, while the IG also outperformed the CG in blood pressure reduction. In multivariable regression, consultation frequency remained a significant predictor of improvement in the number of MetS diagnostic criteria.

Given that consultation emerged as a consistent predictor of improvement across multiple outcomes, a plausible explanatory mechanism is enhanced adherence to dietary recommendations. Although nutritional interventions have been shown to improve MetS components, adherence to dietary change in adults with MetS is often challenging in routine practice. Randomized evidence from the Prevention with Mediterranean Diet-Plus (PREDIMED-Plus) trial demonstrates that an intensive, behaviorally supported, energy-reduced Mediterranean diet program increases diet adherence at 12 months [33]. In our study, the changes in dietary intake patterns observed in the intervention groups support the interpretation that increasing follow-up frequency within MNT can improve dietary nutrient intake. Correlation analyses showed small-magnitude associations between consultation frequency and nutrient intakes, which is expected for multifactorial behavioral outcomes. While PREDIMED-Plus operationalized adherence using a Mediterranean diet score and we assessed intake via 24 h recalls, both approaches converge on the same conclusion—more frequent, structured follow-up is associated with better dietary adherence and cardiometabolic outcomes. Although participants in the intervention groups received nutritional counseling and were encouraged to follow MNT, compliance was influenced by multiple factors. Adherence difficulties to the program were reported by certain participants, primarily in relation to disruptive life events such as bereavement or changes in employment circumstances. Moreover, the earthquake that struck Türkiye on 6 February 2023—one of the most devastating natural disasters in the country’s history—was cited by intervention participants as a major source of emotional distress and reduced dietary adherence. These findings highlight the importance of considering psychosocial and environmental disruptions when evaluating lifestyle interventions.

Beyond traditional face-to-face counseling, the recent literature emphasizes that telehealth-supported MNT may help sustain adherence and continuity of care, particularly for individuals with chronic cardiometabolic conditions. Systematic reviews examining telehealth applications, whose use expanded markedly during the COVID-19 pandemic, show that remote nutrition counseling enhances treatment adherence, supports continuity of care, and improves clinical outcomes in the management of non-communicable chronic diseases. In cardiometabolic conditions such as diabetes and hypertension, telehealth provides a sustainable follow-up mechanism that facilitates behavior change [34,35]. Similar findings have been reported in other systematic reviews, which demonstrate that more frequent contact with hybrid modalities is associated with greater improvements in weight loss, lipid profiles, and blood pressure control. Similarly, analyses from the U.S. CDC’s National Diabetes Prevention Program have shown a graded dose–response relationship between session attendance and cardiometabolic improvements [36].

A pilot randomized controlled trial among overweight and obese adults reported that combining telemonitoring and telehealth coaching with dietary interventions improved nutrition knowledge and was associated with reductions in BMI and visceral fat percentage [37]. Previous pilot studies have suggested that integrating telephone-delivered health coaching into standard multidisciplinary weight management programs can enhance patient engagement and improve weight-related outcomes. For example, Driscoll et al. reported that adults receiving telephone coaching alongside usual care achieved greater reductions in body weight and BMI compared to usual care alone, and participants highlighted the convenience and motivational support provided by remote coaching [38]. Although the study was limited by a small sample size and non-randomized design, these findings align with the present study, supporting the potential of telehealth integration to reinforce adherence to MNT and to optimize cardiometabolic outcomes. Suhlrie et al. reported that participants who received more frequent telehealth-based follow-up achieved superior glycemic control and weight management compared with those receiving standard care [39]. Aligned with this body of evidence, our intensive arm (IIG) demonstrated the largest improvements in body weight, BMI, WC, fat mass, and several cardiometabolic biochemical markers.

This study has several limitations that should be considered when interpreting the findings. First, the follow-up period was limited to three months, which restricts our ability to evaluate the long-term sustainability of behavioral change. Evidence from large international lifestyle trials, such as PREDIMED-Plus, shows that adherence to nutrition therapy often declines over time, underscoring the need for extended monitoring in future research [33]. Second, adherence to MNT was not assessed using a standardized metric. Similarly to many telehealth-based nutrition studies, reliance on self-reported behaviors may have contributed to variability in treatment responsiveness, as some participants may adhere well even with limited contact while others may require more structured support. Measurement-related factors also warrant attention. Anthropometric, biochemical, and blood-pressure values are subject to diurnal variation, and 24 h dietary recalls are inherently prone to recall and reporting bias—limitations commonly reported in metabolic and telehealth literature. The intervention was delivered by a single, unblinded dietitian, which may have introduced performance bias. Additionally, participants were not stratified by baseline MetS severity, a factor known to influence clinical response in international cardiometabolic research. Overall, these limitations highlight the need for longer-term trials incorporating objective adherence assessments, stratification by disease severity, and standardized measurement protocols to strengthen internal validity and enhance the generalizability of future findings.

### Perspectives for Clinical and Assistive Practice

Findings from the present trial indicate that increasing the frequency of dietitian–patient contact together with telehealth meaningfully improves cardiometabolic outcomes in individuals with MetS. Telehealth enables regular follow-up without imposing additional burden on healthcare resources, thereby facilitating more consistent adherence to MNT.

From a clinical standpoint, these results highlight the practicality of hybrid care models that integrate both in-person visits and remote follow-ups. Such approaches allow clinicians to maintain continuity of care, engage patients more effectively, and tailor MNT to individual needs. Evidence from simulation-based statistical modeling further suggests that structured tele-nutrition interventions may sustain benefits over longer horizons, predicting continued improvements in diabetes and hypertension management at one, three, and five years [40]. These projections reinforce the clinical value of the intensified follow-up strategy tested in the current study.

Beyond clinical outcomes, telehealth provides additional system-level advantages. Marković et al. emphasized that telehealth not only offers metabolic benefits but also contributes to environmental sustainability by reducing transportation-related emissions [41]. Likewise, tele-nutrition can help overcome barriers such as geographical distance, limited clinic availability, or time constraints, enabling broader and more equitable access to MNT [34]. Strengthening the integration of telehealth into national health policies and investing in the digitalization of nutrition services could therefore enhance chronic disease management strategies, particularly for MetS.

Ensuring quality in telehealth delivery requires the monitoring of performance indicators. The Telemechron initiative underscores the importance of evaluating patient outcomes, accessibility, and service efficiency to optimize the sustainability of telehealth interventions [42]. Future telehealth studies should incorporate such indicators to support the development of scalable and generalizable care models. Recent reviews also point to the need for structured implementation frameworks that address data security, patient safety, and long-term monitoring as critical priorities for telemedicine in chronic disease care [34,43]. These considerations align with the hybrid telehealth approach used in the present study, underscoring its potential to enhance patient engagement and support long-term cardiometabolic improvement.

Taken together, the literature shows that the well-established advantages of telehealth—greater accessibility, time efficiency, and improved adherence—likely contribute to the mechanisms underlying the positive outcomes observed in this trial [34,35].

## 5. Conclusions

This study demonstrates that increasing the frequency of MNT, particularly when supported by telehealth, leads to clinically meaningful improvements across multiple components of MetS, including WC, body weight, BMI, glycemic indices, and blood pressure. The IIG exhibited the greatest improvements, while even modest increases in counseling frequency (IG) provided significant cardiometabolic benefits, highlighting the potential of scalable, lower-intensity models in routine practice.

These findings emphasize the value of integrating telehealth into dietary counseling to enhance patient adherence, support continuity of care, and optimize long-term outcomes in adults with MetS. Considering the rising prevalence of MetS globally, especially in resource-limited settings, frequent, structured follow-up represents a practical, evidence-based strategy to improve cardiometabolic health. Future studies should further explore the long-term effectiveness and implementation of hybrid MNT models to guide clinical practice and public health interventions.

## Figures and Tables

**Table 1:** The demographic and lifestyle characteristics of participants.

Variables	Intervention Group (*n* = 31)	Intensive Intervention Group (*n* = 30)	Control Group (*n* = 32)	Total (*n* = 93)	*p*-Value ^a^
Age (years) ^b^	46.68 ± 10.4	45.9 ± 11.3	48.8 ± 11.3	47.10 ± 10.88	0.592
Gender ^c^					
Female	20 (64.5)	17 (56.7)	16 (50)	54 (57)	0.508
Male	11 (35.5)	13 (43.3)	16 (50)	39 (43)	
Education status ^c^					
Lower secondary education and below	12 (38.7)	9 (30)	16 (50)	37 (39.8)	0.271
Upper secondary education and above	19 (61.3)	21 (70)	16 (50)	56 (60.2)	
Smoking ^c^	10 (33.3)	10 (34.5)	9 (28.1)	29 (31.9)	0.849
Alcohol usage ^c^	7 (22.6)	5 (16.6)	7 (21.9)	19 (20.4)	0.637
Physical activity level ^c^					
Sedentary	22 (71.9)	26 (86.7)	27 (84.4)	75 (80.7)	0.152
Active	9 (28.1)	4 (13.3)	5 (15.6)	18 (19.4)	
Number of main meals per day ^c^					
≤2	22 (71)	17 (56.6)	21 (65.6)	60 (64.5)	0.5
3	9 (29)	13 (43.3)	11 (34.4)	33 (35.5)	

^a^ < 0.05 significance level. ^b^ Compared with ANOVA method. ^c^ Chi-square analysis was performed.

**Table 2:** Correlations and regression analyses between the number of nutritional counseling sessions and changes in dietary, anthropometric, and biochemical parameters.

Parameters		Number of Nutritional Counseling		
Dietary intake		r ^a^	*p* ^b^	95% CI
	Dietary carbohydrate intake (gr)	−0.394	<0.001 ^b^	[−0.618–0.299]
	Dietary percentage of energy from carbohydrate (%)	0.059	0.576	[−0.147–0.259]
	Dietary protein intake (gr)	−0.094	0.373	[−0.292–0.112]
	Dietary percentage of energy from protein (%)	0.316	0.002 ^b^	[0.120–0.288]
	Dietary fat intake (gr)	−0.178	0.087	[−0.369–0.026]
	Dietary percentage of energy from fat (%)	0.244	0.019 ^b^	[0.042–0.426]
Anthropometric measurements				
	Change in body weight (%)	0.325	0.001 ^b^	[0.130–0.496]
	Change in BMI (kg/m^2^)	0.233	0.025 ^b^	[−0.084–0.317]
	Change in waist circumference (cm)	0.299	<0.001 ^b^	[0.102–0.474]
Biochemical parameters				
	Change in fasting plasma glucose (mg/dL)	0.251	0.015 ^b^	[0.050–0.433]
	Change in insulin (%)	0.213	0.040 ^b^	[0.010–0.399]
	Change in HDL-C (mg/dL)	−0.181	0.082	[−0.371–0.023]
	Change in triglyceride (mg/dL)	0.177	0.089	[−0.027–0.368]
	Change in systolic blood pressure (mmHg)	0.292	0.005 ^b^	[0.094–0.468]
	Change in diastolic blood pressure (mmHg)	0.299	0.004 ^b^	[0.102–0.474]

^a^ Pearson Correlation Coefficient Test was used for parametric data in the table. ^b^ *p* < 0.05 was considered statistically significant. Abbreviations: BMI—body mass index; HDL-C—high density lipoprotein cholesterol.

**Table 3:** Multivariate linear regression analysis of the association between number of nutrition counseling sessions and changes in cardiometabolic parameters.

Regression Model ^a^	B	*p* ^b^	95% CI
Number of nutrition counseling	0.051	0.022 ^b^	0.008–0.095
Change in waist circumference (cm)	−0.065	0.003 ^b^	−0.107–−0.023
Change in body weight (kg)	0.019	0.532	−0.042–−0.080
Change in HDL-C (%)	−0.017	0.003 ^b^	−0.029–0.006
Change in systolic blood pressure (%)	0.020	0.027 ^b^	0.002–0.038
F = 9.395; *p* < 0.001 R = 0.592, R^2^ = 0.351 Adjusted R^2^ = 0.313			

^a^ Multivariate linear regression analysis was performed using backward selection. ^b^ *p* < 0.05 was considered statistically significant. Abbreviations: HDL-C—high density lipoprotein cholesterol.

## Data Availability

The data can be obtained from the corresponding author upon request.

## Abbreviations

The following abbreviations are used in this manuscript:

BMI	Body Mass Index
CG	Control Group
FPG	Fasting Plasma Glucose
IG	Intervention Group
IIG	Intensive Intervention Group
MetS	Metabolic Syndrome
MNT	Medical Nutrition Therapy
MoH	Ministry of Health
MUFA	Monounsaturated Fatty Acid
PUFA	Polyunsaturated Fatty Acid
SFA	Saturated Fatty Acid
PREDIMED	Prevention with Mediterranean Diet
TG	Triglyceride
WC	Waist Circumference
WHO	Word Health Organization

## Appendix A

**Table A1:** Procedures conducted with participants during the interviews.

	Week 0	Week 1	Week 2	Week 3	Week 4	Week 5	Week 6	Week 7	Week 8	Week 9	Week 10	Week 11	Week 12
Interview method	In person	Phone	Phone	Phone	In person	Phone	Phone	Phone	In person	Phone	Phone	Phone	In person
Providing a standard nutrition and physical activity brochure by the physician (TR MoH Healthy Nutrition Brochure)	CG, IG, IIG												
Referral of the patient to the study dietitian	CG, IG, IIG												
Informing participants about the study, obtaining written consent, explaining study rules	CG, IG, IIG												
Recording general characteristics, disease, and family history	CG, IG, IIG												
Assessing dietary habits and applying a food frequency questionnaire	CG, IG, IIG												C, IG, IIG
Determining physical activity levels	CG, IG, IIG				IG, IIG				IG, IIG				C, IG, IIG
Recording a 1-day food intake (Reminder method)	CG, IG, IIG	IIG	IIG	IIG	IG, IIG	IIG	IIG	IIG	IG, IIG	IIG	IIG	IIG	C, IG, IIG
Providing nutrition education based on TR MoH Healthy Nutrition Brochure	CG, IG, IIG												
Preparing an individualized medical nutrition therapy plan	IG, IIG				IG, IIG (if necessary ^a^)				IG, IIG (if necessary ^a^)				
Assessing and encouraging diet adherence		IIG	IIG	IIG		IIG	IIG	IIG		IIG	IIG	IIG	
Anthropometric measurements	CG, IG, IIG				IG, IIG				IG, IIG				C, IG, IIG
Blood pressure measurement	CG, IG, IIG				IG, IIG				IG, IIG				C, IG, IIG
Biochemical measurements	CG, IG, IIG				IG, IIG				IG, IIG				C, IG, IIG

^a^ A novel medical nutrition plan was developed in alignment with the revised energy requirements, contingent upon a variation of ±100 kcal/day.

**Table A2:** Evaluation of changes in energy, fiber, macro and nutrient consumption during week 0, 4, 8 and 12 according to group.

Nutrients	Time	Intervention Group (IG) (*n* = 31)		Intensive Intervention Group (IIG) (*n* = 30)		Control Group (CG) (*n* = 32)		Within-Group *p* *	Between-Group *p* *	F (df_1_, df_2_), *p* *, η^2^
		Mean ± SS	Median	Mean ±SS	Median	Mean ± SS	Median			
			(Min–Max)		(Min–Max)		(Min–Max)			
Energy (kcal)	Baseline	2242.4 ± 774.4	2144.2 (1093.9–3723.9)	2250.4 ± 574.3	2140.85 (1389–3583)	2066.3 ± 648.5	2082.5 (1041–3608.9)	IG: **0.02** ^a^ **w_0_ > w_4_, w_8_, w_12_** IIG: **0.001** ^a^ **w_0_ > w_4_, w_8_, w_12_** CG: 0.06 ^c^	0.47 ^e^	**F (2.4, 140.8)** ***p* < 0.001** ^a^ **η^2^ = 0.371**
	Week 4	1795.8 ± 514.6	1709.6 (1035–3091.6)	1709.2 ± 464.4	1598.3 (1048.1–2900.7)	**–**	**–**		–	
	Week 8	1791.7 ± 505.1	1736.8 (869.4–3027.4)	1602.7 ± 397.7	1527.4 (1041.8–2508.5)	–	–		–	
	Week 12	1704.6 ± 591.3	1742.3 (1888.5–2780)	1586.5 ± 405.4	1530.7 (922.4–2398.1)	2066.6 ± 578	2163.95 (1128.8–3334.9)		**0.02** ^e^ **CG > IG, IIG**	
Carbohydrate (gr/day)	Baseline	221.2 ± 104.6	204 (44–432.8)	201.7 ± 82.5	177.3 (73–408.8)	197.4 ± 77.6	188.7 (89.1–403.9)	IG: **<0.001** ^a^ **w_0_ > w_4_, w_8_, w_12_** IIG: **<0.001** ^a^ **w_0_ > w_4_, w_8_, w_12_** CG: **0.02** ^c^	0.532	**F (2, 115)** ***p* < 0.001** ^a^ **η^2^ = 0.390**
	Week 4	146 ± 55.2	148 (36.9–248.7)	121.5 ± 46.7	121.2 (32.6–238.8)	–	–		–	
	Week 8	145.3 ± 54.0	127 (60.7–242.5)	122.8 ± 35.4	122.9 (66.3–198.1)	–	–		–	
	Week 12	138.5 ± 55.7	124.3 (42.6–240.3)	125.6 ± 41.3	120 (61.7–228.6)	170.5 ± 63.5	165.1 (37.6–308.3)		**0.005** ^e^ **CG > IG, IIG**	
Carbohydrate (%)	Baseline	40.8 ± 7.7	40 (24–58)	37.6 ± 7.7	38 (23–52)	39.7 ± 8.6	38 (26–58)	IG: **0.004** ^a^ **w_0_, w_4_ > w_8_, w_12_** IIG: **<0.001** ^a^ **w_0_ > w_4_, w_8_, w_12_** CG: **0.01** ^c^	0.29	0.076 ^b^
	Week 4	39.3 ± 35.1	34 (12–222)	29.2 ± 8.7	29 (12–49)	–	–		–	
	Week 8	33.4 ± 8.8	33 (17–49)	31.4 ± 5.4	31 (23–41)	–	–		–	
	Week 12	33.2 ± 8.8	34 (17–49)	32.5 ± 7.2	30.5 (22–47)	34.6 ± 9.9	33 (13–58)		0.619 ^e^	
Protein (gr/day)	Baseline	77.0 ± 25.9	76.4 (31.5–126.4)	79.2 ± 23.4	77.7 (42.8–143.8)	77.5 ± 24.5	80 (30.4–116.9)	IG: 0.655 ^a^ IIG: 0.468 ^a^ CG: 0.089 ^c^	0.936 ^e^	0.751
	Week 4	82.4 ± 32.7	78.5 (38.4–186.6)	76.1 ± 22.4	74.1 (32.9–116.3)	–	–		–	
	Week 8	82.5 ± 30.9	75.3 (42.4–175.5)	76.1 ± 24	72.15 (33–123.5)	–	–		–	
	Week 12	78.2 ± 32.2	68.9 (27.2–146.7)	73.62 ± 24.8	71.8 (35.7–125.2)	88.1 ± 31.6	90.9 (25.9–153)		0.149 ^e^	
Protein (%)	Baseline	15.74 ± 7.7	14 (10–32)	15 ± 3.11	14 (11–23)	15.8 ± 4.5	15.5 (10–28)	IG: 0.366 ^b^ IIG: **<0.001** ^b^ **w_12_ > w_0_** CG: 0.119 ^d^	0.712 ^f^	**F (2.6, 150.7)** ***p* = 0.002** ^a^ **η^2^ = 0.091**
	Week 4	18.9 ± 5.4	19 (11–40)	18.7 ± 4.9	18 (11–30)	–	–		–	
	Week 8	18.9 ± 4.4	19 (12–32)	19.6 ± 4.5	19 (10–28)	–	–		–	
	Week 12	18.7 ± 4.3	18 (12–27)	21.2 ± 12.9	19 (11–86)	17.7 ± 5.4	17 (10–33)		0.43 ^f^	
Fat (gr)	Baseline	113.1 ± 37.4	104.5 (54.3–181.6)	123.8 ± 34.5	118 (71.2–202.1)	105.2 ± 40	98.75 (49.2–226)	IG: **0.044** ^a^ **w_0_ > w_4_, w_8_, w_12_** IIG: **<0.001** ^a^ **w_0_ > w_4_, w_8_, w_12_** CG: 0.167 ^c^	0.148 ^e^	**F (3, 2)** ***p* = 0.001** ^a^ **η^2^ = 0.223**
	Week 4	95.1 ± 31.4	86.6 (48.8–165.4)	98.1 ± 36.6	92 (31.6–197.8)	–	–		–	
	Week 8	95.9 ± 36.0	88.5 (41–205.5)	88.3 ± 25.3	79.5 (46.5–141.8)	–	–		–	
	Week 12	92.6 ± 34.7	82.9 (42.9–166.7)	86.7 ± 26.6	80.45 (45.9–142.9)	112.1 ± 37.7	110.1 (41.9–183.5)		**0.009** ^e^ **CG > IG, IIG**	
Fat (%)	Baseline	45.4 ± 5.6	45 (33–54)	48.9 ± 9.9	47.5 (29–68)	45.2 ± 7.3	46 (27–58)	IG: 0.173 ^a^ IIG: 0.199 ^a^ CG: **0.013** ^c^	0.114 ^e^	0.120
	Week 4	48.4 ± 7.7	50 (33–67)	52 ± 7.9	51.5 (38–64)	–	–		–	
	Week 8	47.7 ± 8.9	48 (21–63)	48.9 ± 5.3	50 (34–58)	–	–		–	
	Week 12	48.1 ± 7.2	50 (33–59)	48.6 ± 7.9	48.5 (29–62)	48.2 ± 7.4	48.5 (32–68)		0.954 ^e^	
MUFA (gr)	Baseline	43.7 ± 15.5	44 (15.9–72.5)	44.8 ± 15.5	46.4 (16.1–77)	42.8 ± 21.9	34.8 (22.4–124)	IG: 0.171 ^a^ IIG: **0.018** ^a^ **w_0_ > w_4_, w_8_, w_12_** CG: 0.654 ^c^	0.81 ^e^	**F (3, 8)** ***p* < 0.001** ^a^ **η^2^ = 0.112**
	Week 4	38.5 ± 12.7	36.6 (20.6–67.7)	40.1 ± 13.4	39.9 (17.2–78.3)	–	–		–	
	Week 8	38.4 ± 15.3	32.4 (15.5–78.7)	35.1 ± 11.5	34.1 (4.5–59.9)	–	–		–	
	Week 12	35.8 ± 12	33.7 (12.5–63.8)	34.8 ± 13.6	31.6 (16.1–75.8)	44.6 ± 14.7	43.9 (15.7–79.3)		**0.** **009** ^e^ **CG > IG, IIG**	
SFA (gr)	Baseline	42.27 ± 15.7	37.1 (17.7–73.5)	47.5 ± 19.0	44.4 (11.1–91.1)	40.5 ± 18.0	35.7 (16.7–97.6)	IG: 0.331 ^a^ IIG: **0.005** ^a^ **w_0_ > w_4_, w_8_, w_12_** CG: 0.331 ^c^	0.275 ^e^	**F (1.9, 110.6)** ***p* = 0.018** ^a^ **η^2^ = 0.068**
	Week 4	43.2 ± 36.4	36.2 (12.2–224)	38.4 ± 18	32.7 (19–93.1)	–	–		–	
	Week 8	37.1 ± 15.2	34.8 (13.8–85.3)	33.7 ± 11.4	30.2 (18.4–60.5)	–	–		–	
	Week 12	37.0 ± 16.4	34.6 (7.1–70.6)	33.4 ± 10.4	33.1 (7.4–50.4)	42.8 ± 17.8	41 (14.4–80.2)		0.057 ^e^	
PUFA (gr)	Baseline	19.4 ± 12.6	16.6 (7.8–70.1)	19.5 ± 11.1	17.6 (3–60.3)	16.6 ± 7.8	15.3 (5.7–34.9)	IG: 0.051 ^b^ IIG: **0.009** ^b^ **w_0_ > w_4_, w_8_, w_12_** CG: 0.48 ^d^	0.478 ^f^	**F (1.9, 107)** ***p* = 0.005** ^a^ **η^2^ = 0.071**
	Week 4	14.9 ± 6.6	13.3 (5–29.2)	13.9 ± 6.2	13.6 (4.7–33.1)	–	–		–	
	Week 8	14 ± 7.5	12.2 (4–32.1)	11.9 ± 5.1	11.3 (3.6–21.7)	–	–		–	
	Week 12	18.7 ± 27.6	13.8 (5.6–61.6)	12.5 ± 6.1	11 (1.9–26.1)	16.2 ± 8	14.2 (0.4–35.3)		0.365 ^f^	
Fiber (gr)	Baseline	27.6 ± 10.6	16.5 (7.1–35.7)	22.8 ± 8.7	14.9 (6.1–33.7)	26.4 ± 9.6	15.3 (7.1–33.7)	IG: 0.137 ^a^ IIG: 0.535 ^a^ CG: 0.741 ^c^	0.137 ^e^	0.535 ^b^
	Week 4	25.7 ± 12	11.3 (2.7–20.7)	23.4 ± 7.3	9.4 (2.6–18.6)	–	–		–	
	Week 8	26.5 ± 9.4	10.6 (4.3–19.7)	25.2 ± 9.8	9.5 (5.3–15)	**–**	**–**		**–**	
	Week 12	23.4 ± 10.1	10.5 (3.2–19.9)	24 ± 9.3	9.5 (4.5–19.1)	25.7 ± 7.7	12.7 (2.9–25.7)		0.574 ^e^	
Vit A(µg)	Baseline	1855.9 ± 1256.6	1439.4 (412.5–5663.8)	2550. ± 4080.1	1809.6 (359.4–2608)	1752.4 ± 1277.6	1390.7 (462.3–5776.1)	IG: 0.212 ^b^ IIG: 0.053 ^b^ CG: 0.443 ^d^	0.398 ^f^	0.535 ^b^
	Week 4	2499.6 ± 1672.7	1945.8 (441.6–6134)	2777.2 ± 1915	2527.4 (744.8–9125)	–	–		–	
	Week 8	1846.3 ± 1374.8	1485.8 (617.4–5518.6)	1811.3 ± 1172.1	1485.7 (427.3–4461)	–	–		–	
	Week 12	2005.3 ± 1258	1636.4 (624.3–5437.5)	2906.7 ± 4540.1	1516 (204.3–2424)	1978.9 ± 1511.5	1360 (363.5–5933.1)		0.348 ^f^	
Calcium (mg)	Baseline	849.5 ± 362.3	746.3 (262.8–1499.2)	780.7 ± 303.8	705.1 (384.2–1770.5)	786.4 ± 353.4	734.5 (205.6–1708.9)	IG: 0.55 ^b^ IIG: 0.184 ^b^ CG: 0.286 ^d^	0.68 ^f^	0.184 ^b^
	Week 4	877.9 ± 373.6	947.2 (148.5–1939.8)	950.6 ± 417	857.9 (433.1–2154.7)	–	–		–	
	Week 8	779.9 ± 282.4	758.2 (81–1271)	858.3 ± 357.9	805.3 (278.7–1573)	–	–		–	
	Week 12	904 ± 487.8	777.3 (256.2–3020)	899.1 ± 315.3	957.8 (189.3–1614.7)	860.2 ± 307.2	771.4 (279.7–1446.1)		0.881 ^f^	
Cholesterol (mg)	Baseline	343.2 ± 179.1	336 (45.3–794.4)	329.7 ± 149.6	337 (58.8–781.4)	366.2 ± 251.6	322 (44.8–967.6)	IG: 0.243 ^a^ IIG: 0.065 ^a^ CG: 0.087 ^c^	0.766 ^e^	**F (3, 8)** ***p* = 0.031** ^a^ **η^2^ = 0.049**
	Week 4	429.1 ± 238.8	383.1 (162.4–1014.4)	451.2 ± 206.2	441.3 (168.2–856)	–	–		–	
	Week 8	429.9 ± 294.3	385.4 (51.8–1399)	431.8 ± 213.3	406.2 (52.5–855.2)	–	–		–	
	Week 12	442 ± 291	442 (182–1065.4)	357.4 ± 163.1	327.65 (138–744.6)	459.1 ± 242.9	478.2 (150–983.7)		0.209 ^e^	
Iron (mg)	Baseline	14 ± 5.4	13 (4.9–31.6)	11.9 ± 3.5	11.9 (5.1–20.3)	13.3 ± 4.4	12 (6.3–31.6)	IG: 0.805 ^b^ IIG: 0.556 ^b^ CG: 0.231 ^c^	0.313 ^f^	0.315 ^b^
	Week 4	16.6 ± 8.6	15.4 (7.3–20.3)	13.3 ± 4.7	13.8 (7.2–27.2)	–	–		–	
	Week 8	134 ± 4.1	13.9 (7.6–26.1)	12.3 ± 3.6	11.8 (7.5–20.6)	–	–		–	
	Week 12	14 ± 6.1	12.1 (6.2–28.2)	12.9 ± 5.3	11.2 (5.1–26.7)	15.1 ± 6.1	14.2 (7.4–35.9)		0.355 ^f^	

* Statistical significance was set at *p* < 0.05. Statistical tests used: ^a^ Repeated-measures ANOVA was used. ^b^ Freidman test method was applied. ^c^ Paired sample *t*-test was used. ^d^ The Mann–Whitney U test was conducted. ^e^ One way ANOVA was used. For significant ANOVA results, the differences between specific measurements were determined using the Bonferroni method. ^f^ Kruskal–Wallis analysis was used. For significant results from the Friedman analysis, the Wilcoxon signed-rank test was applied to identify significant differences between the measurements.

**Table A3:** Evaluation of anthropometric measurements during week 0, 4, 8 and 12 according to group.

Anthropometric Measurements	Time	Intervention Group (IG) (*n* = 31)		Intensive Intervention Group (IIG) (*n* = 30)		Control Group (CG) (*n* = 32)		Within-Group *p* *	Between-Group *p* ^b^	F (df_1_, df_2_), *p* *, η^2^
		Mean ± SS	Median	Mean ± SS	Median	Mean ± SS	Median			
			(Min–Max)		(Min–Max)		(Min–Max)			
Body weight (kg)	Baseline	96.2 ± 15.6	94.1 (69.1–131.7)	96.8 ± 17.8	93.6 (61.6–152.9)	89.7 ± 18.3	86.9 (57.5–130.9)	IG: **<0.001** ^a^ **w_0_ > w_4_, w_8_, w_12_** IIG: **<0.001** ^a^ **w_0_ > w_4_, w_8_, w_12_** CG: **0.002** ^c^	**0.002 ^d^**	0.206
	Week 4	93.6 ± 14.8	92 (67.2–124.5)	93.6 ± 17.3	90.1 (61–150.8)	–	–		–	
	Week 8	92.8 ± 14.6	91.4 (66.4–123.1)	92.7 ± 17.3	90.45 (59.4–150.4)	–	–		–	
	Week 12	91.8 ± 14.3	91.1 (66.5–119.8)	92.1 ± 17.1	92.4 (58.5–145.9)	88.3 ± 17.5	85 (55.7–124.6)		0.594**^d^**	
Change in body weight	Baseline—week 12	4.31 ± 3.92	3.9 (−1.3–16.6)	4.7 ± 3.4	4.7 ± 3.4	1.37 ± 1.1	2.3 (−2.3–6.5)	**–**	**<0.001** **^d^** **IG, IIG > CG**	**F (1.5, 91.3)** ***p*** **< 0.001** **η^2^** **= 0.291**
BMI	Baseline	35.3 ± 5.8	35.7 (19.0–45.7)	35.1 ± 4.8	35.8 (24.6–43.1)	33.6 ± 5.3	32.3 (25.2–49.3)	IG: 0.058 ^a^ IIG: **<0.001** ^a^ **w_4_, w_8_ > w_0_, w_12_** CG: 0.121 ^c^	0.392**^d^**	
	Week 4	34.8 ± 4.9	34.6 (25.3–44.6)	36 ± 7.0	34.6 (24.4–40.)	–	–		–	
	Week 8	36.0 ± 9.8	34.1 (25–42.1)	35.7 ± 7.3	33.9 (24.4–40.1)	–	–		–	
	Week 12	35.7 ± 9.9	33.5 (25–42.2)	33.4 ± 4.7	33.8 (23.2–40)	33 ± 5.1	32 (24.4–49)		0.268	
Change in BMI	Baseline—week 12	1.2 ± 2.5	1.3 (−10.16–4.5)	1.7 ± 1.3	1.9 (−2.4–3.6)	0.6 ± 0.9	0.45 (−0.8–2.8)	**–**	**0.** **001 ^d^** **IG, IIG > CG**	
Body fat (%)	Baseline	43.2 ± 9	43.7 (28.8–59.8)	43.1 ± 9.6	47.7 (22.3–56.4)	41.8 ± 6.6	43.7 (29.6–51)	IG: **0.001** ^a^ **w_0_, w_4_ > w_8_, w_12_** IIG: **<0.001** ^a^ **w_0_ > w_4_, w_8_, w_12_** CG: **0.003** ^c^	0.78**^d^**	**F (1.7, 101.9)** ***p*** **= 0.001** **η^2^** **= 0.120**
	Week 4	43.1 ± 9	45.3 (27.7–59.5)	41.9 ± 9.3	45.8 (22.3–54.8)	–	–		–	
	Week 8	41.3 ± 10.3	44.6 (13.3–56.5)	41.2 ± 9.7	44.3 (21.2–54.1)	–	–		–	
	Week 12	41.9 ± 8.9	44.8 (25.4–56.9)	40.7 ± 9.6	43.1 (21.2–55.6)	39.1 ± 6.9	39 (24.7–49.5)		0.377 **^d^**	
Change in body fat	Baseline—week 12	1.3 ± 1.9	1.4 (−2.9–5.2)	2.4 ± 2.5	1.95 (−3.5–9.3)	2.69 ± 2	4.7 (−7.7–14)	**–**	0. 269**^d^**	
FFM (%)	Baseline	25.6 ± 4.4	25.1 (17.7–33.3)	26.4 ± 4.9	24.6 (18.7–36.8)	25.8 ± 3.4	25.1 (21.3–32.1)	IG: 0.052 ^a^ IIG: **0.004** ^a^ **w_8_, w_12_ > w_0_, w_4_**, CG: **<0.001** ^c^	0.773**^d^**	**F (2.5, 177)** ***p*** **= 0.002** **η^2^ = 0.087**
	Week 4	25.4 ± 4.4	24 (17.6–33.7)	26.9 ± 4.8	25 (19.9–36.3)	–	–		–	
	Week 8	25.9 ± 4.4	24.9 (19–34)	27.1 ± 5.3	25.1 (19.9–38.1)	–	–		–	
	Week 12	26.1 ± 4.4	24.4 (19.1–34.9)	27.6 ± 5.5	26.5 (19.7–40)	27.2 ± 3.6	27.6 (22.4–34.8)		0.439**^d^**	
Change in FFM	Baseline—week 12	0.5 ± 1.1	0.7 (−2.6–2)	1.3 ± 2.3	1.25 (−5.9–7.8)	1.4 ± −1.8	−1.3 (−5.8–3.6)	**–**	0.103**^d^**	
Waist circumference	Baseline	110.9 ± 11.5	111 (89–134)	111.8 ± 10.4	110.5 (90–135)	108.3 ± 13	105.5 (88–135)	IG: **<0.001** ^a^ **w_0_ > w_4_ > w_8_ > w_12_** IIG: **<0.001** ^a^ **w_0_ > w_4_ > w_8_ > w_12_** CG: **0.921** ^c^	0.475**^d^**	**F (1.8, 107.3)** ***p*** **< 0.001** **η^2^** **= 0.507**
	Week 4	108.2 ± 10.2	108 (87–126)	108.1 ± 9.8	106.5 (88–133)	–	–		–	
	Week 8	106.4 ± 11.4	106 (82–129)	106.4 ± 9.7	108 (84–126)	–	–		–	
	Week 12	104.3 ± 11.1	104 (81–126)	105.4 ± 11.4	104.5 (81–129)	107 ± 11.6	106 (87–128)		0.629**^d^**	
Change in waist circumference	Baseline—week 12	6.6 ± 5.8	6 (−2–28)	6.4 ± 4.6	6 (1–20)	1.3 ± 2.7	1.3 (−5–8)		**<0.001 ^d^** **IG, IIG > CG**	

* Statistical significance was set at *p* < 0.05. Statistical tests used: ^a^ Repeated-measures ANOVA was used. ^b^ Freidman test was used. ^c^ Paired sample *t*-test was used. ^d^ Analysis of variance (ANOVA) was used. For significant ANOVA results, the differences between specific measurements were determined using the Bonferroni method. For significant results from the Friedman analysis, the Wilcoxon signed-rank test was applied to identify significant differences between the measurements. The change values were calculated based on the changes observed between baseline (week 0) and week 12.

**Table A4:** Evaluation of biochemical measurements and blood pressures during week 0, 4, 8, 12 during week 0, 4, 8 and 12 according to groups.

Biochemical Parameters	Time	Intervention Group (IG) (*n* = 31)		Intensive Intervention Group (IIG) (*n* = 30)		Control Group (CG) (*n* = 32)		Within-Group *p **	Between-Group *p* *	F (df_1_, df_2_), *p* *, η^2^
		Mean ± SS	Median	Mean ± SS	Median	Mean ± SS	Median			
			(Min–Max)		(Min–Max)		(Min–Max)			
Fasting plasma glucose (mg/dL)	Baseline	107 ± 16.5	104 (78–135)	119.6 ± 42.8	112 (71–298)	131.4 ± 41	126.5 (83–238)	IG: **0.004** ^a^ **w_0_ > w_4_, w_8_, w_12_** IIG: **<0.001** ^a^ **w_0_ > w_4_, w_8_, w_12_** CG: **0.03** ^c^	**0.021** ^e^ **CG > IG, IIG**	**F (1.3, 79.2)** ***p*** **< 0.001** **η^2^** **= 0.215**
	Week 4	101.8 ± 14.3	99 (78–132)	98.9 ± 12.5	94.5 (83–129)	–	–		–	
	Week 8	98.5 ± 13.1	98 (72–128)	95.9 ± 13.4	93.5 (72–139)	–	–		–	
	Week 12	100.6 ± 12.6	101 (81–130)	97.3 ± 1	92.5 (75–136)	123 ± 112.5	112.5 (82–214)		**0.001** ^e^ **CG > IG, IIG**	
Change in fasting plasma glucose	Baseline—week 12	6.4 ± 9.4	7 (−15–27)	22.2 ± 37.6	19.5 (−19–198)	8.4 ± 23.7	2.5 (−16–113)	–	**0.009** ^e^ **IIG > IG, CG**	
İnsulin (mU/L)	Baseline	15.84 ± 7.81	15.5 (3.1–34.5)	16.9 ± 5.3	15.4 (9.1–33)	14.2 ± 8.6	11.7 (5.6–49.2)	IG: **<0.001** ^a^ **w_0_ > w_4_, w_8_, w_12_** **IIG: 0.001** ^a^ **w_0_ > w_4_, w_8_, w_12_** **CG: 0.036** ^c^	0.135 ^e^	**F (3, 177)** ***p*** **< 0.001** **η^2^** **= 0.168**
	Week 4	14.55 ± 7.62	13.5 (5.1–40.3)	14.2 ± 6.7	13.2 (2.5–30)	–	–		–	
	Week 8	12.02 ± 6.94	9.6 (4.3–32.4)	11.8 ± 4.1	11.5 (2.5–22.3)	–	–		–	
	Week 12	12.47 ± 6.28	10.7 (4.4–25.7)	14 ± 5.8	12.3 (2.4–32.1)	13.1 ± 10.4	10.4 (5.4–45.6)		0.33 ^e^	
Change in insulin	Baseline—week 12	3.37 ± 4.3	2.7 (−3.1–16.7)	2.9 ± 7.1	3.4 (−9.6–24.4)	1.0 ± 2.8	0.35 (−3.4–9.1)	**–**	0.194 ^e^	
HOMA-IR	Baseline	4.0 ± 1.9	4.1 (0.8–7)	4.4 ± 2.0	4 (1.9–10.8)	4.7 ± 2.8	3.4 (1.3–10.3)	IG: **<0.001** ^a^ **w_0_, w_4_ > w_8_, w_12_** IIG: **<0.001** ^a^ **w_0_ > w_4_, w_8_, w_12_** **CG: 0.001** ^c^	0.799 ^e^	**F (1.5, 86.5)** ***p*** **= 0.015** **η^2^ = 0.008**
	Week 4	4.5 ± 5.5	3.3 (1.1–32)	3.6 ± 1.9	3.1 (0.7–9)	–	–		–	
	Week 8	2.9 ± 1.8	2.2 (1.1–8.3)	3.8 ± 5.1	2.7 (1.3–30.4)	–	–		**–**	
	Week 12	3.2 ± 1.8	2.4 (1–7)	3.4 ± 1.4	2.8 (1.7–7.4)	4.1 ± 3.3	3.26 (1.6–9.8)		0.369 ^e^	
Change in HOMA-IR	Baseline—week 12	0.9 ± 1.3	0.9 (−1.3–4.7)	1.1 ± 2	0.6 (−2.2–6.5)	0.6 ± 1.5	0.255 (−1.7–7.3)	**–**	0.479 ^e^	
HbA1c (%)	Baseline	6 ± 0.7	6 (4.9–7.9)	6.1 ± 0.9	6.05 (4.9–8.3)	6.2 ± 0.8	6 (5.4–8.3)	IG: **0.013** ^b^ **w_0_ > w_12_** **IIG: 0.044** ^b^ **w_0_ > w_12_** **CG: 0.043** ^d^	0.63 ^f^	**F (2.1, 112.4)** ***p*** **< 0.001** **η^2^ = 0.161**
	Week 4	5.8 ± 0.6	5.8 (5.1–7.3)	5.9 ± 0.6	5.9 (5.1–7.6)	–	–			
	Week 8	5.7 ± 0.4	5.7 (5.1–6.4)	5.9 ± 0.6	5.7 (4.9–7.4)	–	–			
	Week 12	5.7 ± 0.4	5.6 (5–6.4)	5.8 ± 0.6	5.7 (5.2–7.3)	6.1 ± 6	6 (4.2–7.8)		**0.041** ^e^ **CG > IG, IIG**	
Change in HbA1c	Baseline—week 12	0.4 ± 0.6	0.2 (−0.78–1.6)	0.3 ± 0.6	0.2 (−0.6–1.8)	0.2 ± 0.5	0.1 (−0.6–2.3)	–	0.41 ^e^	
Total cholesterol (mg/dL)	Baseline	224.3 ± 38.8	224 (159–290)	202.8 ± 35.8	204 (132–259)	207.7 ± 42.8	205 (96–310)	IG: **0.012** ^b^ **w_0_ > w_4_, w_8_, w_12_** IIG: 0.167 ^b^ CG: **0.008** ^d^	0.085 ^f^	**F (2.4, 141.7)** ***p*** **< 0.001** **η^2^ = 0.127**
	Week 4	203 ± 43.7	202 (141–311)	189 ± 404	188 (111–303)	–	–			
	Week 8	202.9 ± 38.4	205 (141–291)	190.2 ± 42.1	187.5 (103–304)	–	–			
	Week 12	208.3 ± 37	206 (139–292)	196.7 ± 35.4	204 (115–257)	193.3 ± 195.5	195.5 (101–290)		0.26 ^e^	
Change in total cholesterol	Baseline—week 12	16 ± 41.7	6 (−32–120)	6.1 ± 26.4	2.5 (−38–97)	14.4 ± 28.6	7.5 (−25–121)	–	0.466 ^f^	
HDL-C (mg/dL)	Baseline	46 ± 15.4	43 (25–90)	39.9 ± 7.4	38.5 (32–66)	46.8 ± 14	43.5 (33–86)	IG: 0.894 ^b^ IIG: 0.381 ^b^ CG: 0.543 ^d^	0.098 ^e^	0.381
	Week 4	45.1 ± 13.6	45 (26–82)	41.8 ± 7.5	41 (32–69)	–	–			
	Week 8	45.5 ± 14.7	40 (25–80)	42.7 ± 9	40 (31–66)	–	–			
	Week 12	45.2 ± 13.1	41 (29–79)	42.9 ± 7.4	42 (33–66)	47.8 ± 45	45 (32–85)		0.309 ^f^	
Change in HDL-C	Baseline—week 12	0.8 ± 6.0	−1 (−9–16)	−3.0 ± 6.6	−1.5 (−23–7)	−1.1 ± 6.9	0(−25–13)	–	0.184 ^e^	
LDL-C (mg/dL)	Baseline	146.8 ± 38.1	143 (74–273)	126.8 ± 45.6	127.5 (60–250)	204.3 ± 83.0	193 (52–406)	IG: **0.019** ^a^ **w_0_ > w_4_, w_8_, w_12_** IIG: 0.51 ^a^ CG: 0.51 ^c^	**0.022** ^e^ **IG > IIG, CG**	**F (2.2, 129.5)** ***p*** **= 0.002** **η^2^ =** **0.008**
	Week 4	124 ± 37.3	120 (68–241)	118.4 ± 37	121 (50–228)	–	–		–	
	Week 8	124.6 ± 34.3	127 (50–215)	118.9 ± 42.2	116.5 (21–214)	–	–		–	
	Week 12	132.8 ± 35.2	137 (68–225)	117.4 ± 32.8	127.5 (18–171)	193.9 ± 185	185 (42–480)		0.329 ^e^	
Change in LDL-C	Baseline—week 12	14 ± 41.6	6 (−29–110)	9.4 ± 44.4	1 (−52–131)	10.4 ± 39	5.5 (−86–98)	**–**	0.907 ^e^	
Triglyceride (mg/dL)	Baseline	190.7 ± 55.1	172 (94–346)	232.8 ± 111.9	201.5 (110–633)	204.3 ± 83	193 (52–406)	IG: **0.002** ^a^ **w_0_ > w_4_, w_8_, w_12_** IIG: **<0.001** ^a^ **w_0_ > w_8_, w_12_ > w_4_** CG: **0.042** ^c^	0.383 ^e^	**F (2.2, 129.1)** ***p*** **< 0.001** **η^2^ =** **0.176**
	Week 4	159.2 ± 60.7	151 (86–339)	148.3 ± 51.2	141 (61–240)	–	–		–	
	Week 8	172.5 ± 73.7	157 (65–346)	165.4 ± 63.2	167.5 (68–271)	–	–		–	
	Week 12	162 ± 76.9	143 (60–389)	188.3 ± 88	163 (87–516)	193.9 ± 185	185 (42–480)		0.111 ^e^	
Change in triglyceride	Baseline—week 12	28.7 ± 49.1	31 (−91–129)	44.6 ± 110.3	25 (−195–468)	10.4 ± 39	5.5 (−86–98)	–	0.177 ^f^	
hs-CRP (mg/L)	Baseline	4.5 ± 3.9	3.8 (0.3–16.4)	5.9 ± 5	4.4 (1–19.5)	4.6 ± 3.5	4.5 (0.3–13.8)	IG: 0.381 ^a^ IIG: 0.063 ^a^ CG: 0.604 ^c^	0.458 ^f^	0.189
	Week 4	4.2 ± 4.3	2.75 (0.5–22.8)	3.9 ± 3.7	2.5 (0.5–13.8)	–	–		–	
	Week 8	4.8 ± 7.0	3.1 (0.4–38.8)	5.21 ± 7.9	2.8 (0.6–38)	–	–		–	
	Week 12	4.3 ± 4.8	2.6 (0.6–27)	4.2 ± 5.7	2.1 (0.5–30.4)	4.4 ± 3	3 (0.3–17)		0.707 ^f^	
Change in hs-CRP	Baseline—week 12	0.2 ± 3.8	0.3 (−13.3–10.3)	1.7 ± 5.1	1 (−12.1–17.9)	0.2 ± 3.4	0.02 (−11.5–9.4)		0.246 ^f^	
AST (mg/dL)	Baseline	21.9 ± 7.9	21 (12–40)	22.8 ± 10	20 (12–63)	21.6 ± 8.2	20 (11–49)	IG: 0.324 ^a^ IIG: 0.173 ^a^ CG: 0.909 ^c^	0.964 ^f^	0.127
	Week 4	21 ± 8.9	19 (11–52)	21.6 ± 9.4	19 (12–61)	–	–		–	
	Week 8	20.9 ± 7.6	20 (12–44)	20 ± 5.1	19.5 (9–32)	–	–		–	
	Week 12	20.8 ± 10	18 (11–57)	19.3 ± 4.5	19 (13–36)	22.7 ± 20	20 (11–72)		0.38 ^f^	
Change in AST	Baseline—week 12	1.1 ± 9.3	1 (−38–16)	3.47 ± 9.4	1.5 (−6–45)	−1.1 ± 9	0(−43–11)	–	**0.304**	
ALT (mg/dL)	Baseline	28 ± 18.9	23 (12–100)	29.9 ± 16.5	27 (12–89)	30.1 ± 20.8	24 (12–101)	IG: 0.13 ^b^ IIG: **0.028** ^b^ **w_0_ > w_4_, w_8_, w_12_** CG: 0.315 ^d^	0.566 ^f^	**F (2.2, 131.4)** ***p*** **= 0.004** **η^2^ =** **0.071**
	Week 4	26.1 ± 15.7	23 (11–89)	25.8 ± 12.3	21.5 (13–74)	–	–		–	
	Week 8	25.2 ± 12.4	24 (11.6–61)	23.4 ± 8.5	22.5 (9–55)	–	–		–	
	Week 12	23.7 ± 12.3	19 (11–53)	22.4 ± 8.1	20 (11–44)	28.7 ± 23	23 (13–88)		0.445 ^f^	
Change in ALT	Baseline—week 12	4.3 ± 17.4	3 (−36–78)	7.5 ± 14.3	5 (−14–59)	1.4 ± 20.9	1 (−65–86)	–	0.176 ^f^	
Sistolic Blood Pressure (mmHg)	Baseline	142.5 ± 18.1	136 (120–192)	141 ± 16.5	140.5 (111–168)	144.3 ± 22.4	144.5 (109–192)	IG: **0.015** ^b^ **w_0_ > w_4_, w_8_, w_12_** IIG: **<0.001** ^b^ **w_0_ > w_4_, w_8_, w_12_** CG: 0.113 ^d^	0.791 ^f^	**F (2.1, 121.4)** ***p*** **< 0.001** **η^2^** **= 0.276**
	Week 4	137.3 ± 16.8	134 (112–179)	130.4 ± 15	127 (105–156)	–	–		–	
	Week 8	133.4 ± 18.1	133 (108–194)	126.5 ± 13.8	124 (95–152)	–	–		–	
	Week 12	132.4 ± 18.6	133 (105–176)	124.6 ± 13.8	122.5 (101–152)	140.7 ± 147.5	147.5 (100–167)		**0.002** ^f^ **CG > IG, IIG**	
Change in SBP	Baseline—week 12	10.1 ± 18.3	5 (−22–54)	16.4 ± 16.7	18.5 (−11–50)	3.7 ± 12.8	0(−23–37)	**–**	**0.01** **IIG > IG > CG**	
Diastolic Blood Pressure (mmHg)	Baseline	83.3 ± 15.7	82 (51–133)	86.9 ± 13	87.5 (72–140)	84.9 ± 15.9	82.5 (61–128)	IG: **0.015** ^a^ **w_0_ > w_4_, w_8_, w_12_** IIG: **<0.001** ^a^ **w_0_ > w_4_, w_8_, w_12_** CG: 0.34 ^c^	0.254 ^e^	**F (1.7, 97.6)** ***p*** **< 0.001** **η^2^** **= 0.148**
	Week 4	80.1 ± 9.1	82 (62–97)	81.1 ± 9	80 (64–95)	–	–		–	
	Week 8	79.7 ± 10.32	79 (60–101)	78.3 ± 8.9	79.5 (61–97)	–	–		–	
	Week 12	78.23 ± 10.4	77 (60–103)	77.6 ± 8.2	78 (60–96)	88.3 ± 86	86 (61–129)		**0.005** ^e^ **CG > IG, IIG**	
Change in DBP	Baseline—week 12	5.06 ± 14.59	7 (−22–61)	9.4 ± 13.8	8.5 (−15–65)	−3.4 ± 15.1	0(−46–37)	–	**0.003** ^e^ **IIG > IG > CG**	

* Statistical significance was set at *p* < 0.05. Statistical tests used: ^a^ Repeated-measures ANOVA was used. ^b^ Freidman test was used. ^c^ Paired sample *t*-test was used. ^d^ The Wilcoxon signed-rank test method was applied. ^e^ Analysis of variance (ANOVA) was used. For significant ANOVA results, the differences between specific measurements were determined using the Bonferroni method. For significant results from the Friedman analysis, the Wilcoxon signed-rank test was applied to identify significant differences between the measurements. ^f^ Kruskal–Wallis analysis was used. The change values were calculated based on the changes observed between baseline (week 0) and week 12.
